# IL-7/IL7R axis dysfunction in adults with severe community-acquired pneumonia (CAP): a cross-sectional study

**DOI:** 10.1038/s41598-022-13063-x

**Published:** 2022-07-30

**Authors:** Sandra Ampuero, Guillermo Bahamonde, Fabián Tempio, María Luisa Garmendia, Mauricio Ruiz, Rolando Pizarro, Patricio Rossi, Lucía Huenchur, Luis Lizama, Mercedes López, Luis F. Avendaño, Vivian Luchsinger

**Affiliations:** 1 Programa de Virología, ICBM, Facultad de Medicina, Universidad de Chile, Av. Independencia 1027, Independencia, Santiago, Chile; 2 Programa de Inmunología, ICBM, Facultad de Medicina, Universidad de Chile, Santiago, Chile; 3 Instituto Milenio de Inmunología e Inmunoterapia, Facultad de Medicina, Universidad de Chile, Santiago, Chile; 4 Instituto de Nutrición y Tecnología de los Alimentos, Universidad de Chile, Santiago, Chile; 5 Departamento de Medicina, Hospital Clínico Universidad de Chile, Santiago, Chile; 6 Hospital Lucio Córdova, Santiago, Chile; 7 Complejo Hospitalario San José, Santiago, Chile

**Keywords:** Biomarkers, Medical research

## Abstract

Community-acquired pneumonia (CAP) is a worldwide leading cause of death. Recognized risk factors in some severe cases have not been identified. Lymphocytopenia has been frequently described in CAP. Since IL-7, membrane-bound receptor (IL7Rα;CD127) and soluble IL7Rα (sIL7R) are critical in lymphocytes homeostasis, in this work we aimed to evaluate the involvement of the IL-7/IL7Rα axis in the severity of adult CAP, since it has not been explored. The *IL7R*α SNPs rs6897932, rs987106, and rs3194051 SNPs in *IL7*α were genotyped, the systemic expression of the *IL7R* gene, sIL7R, IL-7, and levels of peripheral IL7Rα^+^ T lymphocytes were quantified in 202 hospitalized CAP cases. rs3194051GG was more frequent in non-survivors than in survivors; rs987106TT was more frequent and rs3194051AA less frequent in patients at intensive care unit (ICU) than in those not admitted to ICU. IL7Rα gene expression was lower in non-survivors than in survivors, and in severe than in mild cases. CD3^+^CD127^+^ lymphocytes were lower in severe than in mild cases; in non-survivors than in survivors and in ICU than in non- ICU admitted cases. sIL7Rα plasmatic levels were higher in non-survivors than in survivors, and in severe than in mild cases. rs6897932CC, rs987106AA and rs3194051GG carriers showed the highest while rs6897932TT showed the lowest sIL7Rα levels. The AUC of sIL7Rα levels predicting 30-day mortality was 0.71. Plasma IL-7 levels were lower in ICU-admitted than in not ICU-admitted and in non-survivors than in survivors. No additional association was detected. In conclusion, rs3194051GG and rs987106TT IL7R genotypes were associated with a poorer prognosis. A significant association between sIL7R levels and SNPs of the IL7R gene is described for the first time in adult CAP. Increased plasmatic sIL7R could contribute to identifying adult CAP cases at risk of death.

## Introduction

Community-acquired pneumonia (CAP) is a frequent worldwide cause of death^1^. In Chile, CAP is the seventh cause of death, particularly among the elderly^2^. Some CAP patients need to be admitted to intensive care unit (ICU) (10–20%)^1^, and some will die (5–10%)^2^. Advanced age and comorbidities (e.g. liver damage, cardiac insufficiency) are known risk factors which are included in clinical scores of severity such as the Pneumonia Severity Index (PSI) and CURB-65^1,3^. However, these factors are not detected in some severe cases, and patients are wrongly classified^4^. A better understanding of the pathogenic factors is crucial to develop early biomarkers which could improve evaluation of severity risk at admission.

The immune response is substantial in the control and recovery of the infection. Lymphocytopenia has been described in CAP, probably related to T-cells and T-cell subsets^5^, and it has even been proposed as a predictive marker^4^. In CAP patients, it is hypothesized that depression of absolute peripheral blood T-cell counts represents the transfer of these cells towards the lung in order to be sequestered by protective mechanisms^5^. However, other factors could also be relevant.

Interleukin 7 (IL-7) plays a critical role in the homeostasis of the immune system, leading to the differentiation, proliferation, and survival of B, T, and natural killer (NK) cells^6^. In addition, patients with mutations in the extracellular domain of IL-7 receptor (IL7Rα or CD127) have severe combined immunodeficiency (SCID) of the T^−^B^+^NK^+^ cells and suffer recurrent and severe respiratory bacterial and viral infections from the early infancy^7^. IL-7 is constitutively produced and released by specialized non-hematopoietic cells from the thymus stroma, bone marrow, liver, spleen, and kidneys^8^. It is locally bound to heparan sulfate proteoglycans, either on the surface of stromal cells or in the extracellular matrix; consequently, levels of IL-7 in human plasma are low, exhibiting values < 10 pg/ml in healthy adults^9,10^.

This cytokine interacts with its unique α-receptor membrane-bound IL7R, forms a ternary complex with common γc receptor and trigger a signaling cascade which facilitates target gene transcription related with anti-apoptotic bcl-2 family^9^. IL7Rα is expressed on the lymphoid lineage and is required during early stage of T cell development and for proliferation and survival of T cells in the periphery^7^. In addition, a soluble IL7Rα (sIL7R or sCD127) is generated for alternative splicing, which provides a modulatory control mechanism regulating extracellular bioavailability of IL-7^6,10^.

Genetic variation in and the expression of the *IL7R* gene, as well as increased expression of receptor/ligand genes, contribute to the pathogenesis of autoimmune and chronic inflammatory diseases such as type 1 diabetes and multiple sclerosis^7^. Single-nucleotide polymorphism (SNP) of *IL7R* gene, such as rs6897932, rs3194051 and rs987106, has been associated with clinical severity in multiple sclerosis, hepatitis C and human immunodeficiency virus infection^11–14^. Four common haplotypes of the *IL7R* gene have been described in exonic and intronic zones and found to be associated with differential levels of sIL7R. This isoform is produced by alternative splicing of the preRNAm that excludes exon 6 (encoding for the transmembrane domain) and generates a stop codon in exon-8 and a soluble protein^12^. Changes in IL-7 bioavailability due to decreased expression of the membrane isoform of IL7Rα and increased soluble isoform levels have been proposed^10^. In addition, high plasma levels of this cytokine have been associated with lethality in sepsis^15^.

Recent studies support a critical role for IL-7 and its receptor in immunosenescence, and while low expression of the IL7Rα in the elderly was associated with a better metabolic profile, higher expression of IL7Rα was linked with protection from mortality^6^. Since age is a known risk factor of CAP severity and the expression of IL7Rα decreases with age, but not IL-7 levels^6^, it is relevant to explore these parameters in adults with CAP. Moreover, since IL-7 therapy could enhance and broaden the immune responses it could be used as severity biomarkers.

The aim of this study was evaluate the association of the components of IL-7/IL7Rα axis with clinical severity in adults with CAP, assessing the allelic and genotypic frequency of certain SNPs, expression of the *IL7R* gene, plasma concentration of soluble IL7R, IL-7 and the proportion of lymphocytes with membrane bound IL7R.

## Methods

### Patients and study design

In this cross-sectional study, 202 patients aged ≥ 18 years old hospitalized for CAP were enrolled in three hospitals (Clínico Universidad de Chile, de Infecciosos Dr. Lucio Córdova Hospital, and Complejo Hospitalario San José) in Santiago, Chile from May 2017 to August 2019. CAP was defined as the presence of acute respiratory symptoms for less than one week plus a chest X-ray display new pulmonary infiltrates^2,4^. Patients with immunocompromising conditions (i.e., organ transplant, active treatment for cancer, human immunodeficiency virus infection, immunosuppressive and, or steroids therapy), hemodialysis and hospitalizations within 30 days preceding enrollment were excluded. Clinical and epidemiological data of the all patients were obtained from medical records, including age, sex, comorbidities (i.e., hypertension; diabetes mellitus; chronic obstructive respiratory disease; chronic liver disease (chronic liver damage suggested by abdominal ultrasound imaging and/or computed tomography scan); chronic kidney damage (serum creatinine level ≥ 2 mg/dL); cardiac insufficiency (heart failure with reduced ejection fraction (≤ 45%)); neurologic disease (nervous system disorders including epilepsy, Parkinson's Disease, Alzheimer's Disease, and stroke); smoking; alcohol consumption (> 80 g per day yes/no); clinical presentation (fever, respiratory symptoms); chest radiographic patterns (consolidation and pleural effusion), and laboratory parameters (i.e., hemogram, plasmatic concentration of glucose, sodium and blood urea nitrogen).

The severity of patients was evaluated according to PSI^3^ and CURB-65^1^ clinical scores applied during the first 48 h after enrollment: IV and V groups of PSI and ≥ 3 of CURB-65 score were considered as severe, while I and II groups of PSI and ≤ 2 of CURB-65 score were considered as mild. Several outcome variables were analyzed: death up to 30 days post- discharge, admission to ICU, use of mechanical ventilation (MV), and supplemental oxygen therapy. Basal values of different parameters were determined in 22 asymptomatic adults without respiratory infection at least 45 days before enrollment and with no pathogen detected in microbiological screening. The study was approved by the Committee on Research in Human Beings of Faculty of Medicine, University of Chile; the Ethics Committee of Clínico U. Chile Hospital, and the Ethics Committees of the South and North Metropolitan Health Service. Written informed consent was provided by all patients at enrolment. The study was performed in accordance with the principles of the Declaration of Helsinki.

### Microbiological study

An immunochromatographic test (Binax NOW, Portland, Oregon, USA) was used to detect *Streptococcus pneumoniae* and *L. pneumophila* serogroup 1 antigens in urine. For the detection of other respiratory pathogens, total genetic material was extracted from nasopharyngeal aspirate/swabs samples (300 µL) by Favorprep™ Viral Nucleic Acid Extraction kit (Favorgen®), according to manufacturer’s instructions, and after was quantified in an EPOCH® spectrophotometer. Human respiratory viruses such as respiratory syncytial virus, influenza A and B viruses, adenovirus, metapneumovirus, bocavirus, coronavirus (HCoV 229E; HCoV OC43, HCoV-NL63, and HCoV-HKU1), parainfluenza virus, rhinovirus/enterovirus, and bacterias (*Chlamydophila pneumonia*e, *Mycoplasma pneumoniae* and *Legionella pneumophila)* were detected by Respiratory Multi well system r-gene Real Time RT-PCR kit Argene®, according to the manufacturer’s instructions in a MIC® thermocycler (BMS®).

### SNPs genotyping

Three single nucleotide polymorphisms located at the *IL7R* gene were evaluated: rs987106 in an intronic region (c.801-21 A > T), rs6897932 in exon 6 (transmembrane region; c.731 C > T; p.Thr244Ile), and rs3194051 in exon 8 (intracellular region; c.1066 A > G; p.Ile356Val).

Only Chilean people were studied. This population is over 90% admixture Caucasian and South amerindian people^16^.

### Extraction of genomic DNA

Genomic DNA was extracted from venous blood (250 μl) with EDTA as anticoagulant using Exgene™ Blood SV kit (GeneAll®) according to the manufacturer instruction. All extracted DNA samples were treated with RNase A (Thermo Scientific, USA) and quantified by spectrophotometry (Epoch (BioTek®). Aliquots (5 ng/μl) were prepared and stored at −20 °C.

### Primers design

Primer sets for the amplifications were designed based on the sequence NG_009567.1 using the software Beacon Designer 8.02 (PREMIER Biosoft, int). The specificity of primers was confirmed on Primer-BLAST (http://www.ncbi.nlm.nih.gov/tools/primer-blast). The primer set for each SNP were: rs6897932: HD: CCACAATCTATTCTTGCTTTCCA and HR: 5´-CCAACAGAGCGACAGAGA-3´; rs987106: 801A-PH: 5´-CTGCCATTCACTTCATCTAT-3´, 801 T-PH: 5´-CTGCCATTCACTTCATCTTT-3´ and 801-PR: 5´-ATGTTCCAGAGTCTTCTTATG-3´; rs3194051: HD: 5´-AACTGCCCATCTGAGGAT-3´ and HR: 5´-GCATGTGAGGGATGAATCT-3´.

### PCR amplification and genotyping

SNPs were independently analyzed. rs6897932 and rs3194051 SNPs were genotyped using in-house PCR-HRM assay validated by direct sequencing. The PCR-HRM reaction was prepared in a final volume (10 μL) which includes DNA template (10 ng), KAPA HRM FAST Master Mix (5 μl) (KAPA HRM FAST kit; Kapabiosystems^©^), MgCl2 (2.5 mM) and each primer set (0.25 μM). Amplifications were performed in an ECO™ Real-Time PCR System Thermocycler (Illumina®). PCR amplifications were performed with initial denaturing at 95 °C for 2 min, and subsequently subjected to 45 cycles: 95 °C for 5 s (denaturalization), 56 °C for 15 s to rs6897932 or 55 °C for 20 s to rs3194051 (annealing and extension). After PCR amplification, the HRM was carried out over the range 55–95 °C rising at 0.1 °C/s. The genotype of each sample was determined by Eco software 4.1.02.0.

Allele-specific PCR was applied for genotyping SNP rs987106, using a specific forward primer to each allele (A or T) and a common reverse primer as described above in “Primer design”. Two reactions were performed for each sample. Final volume (10 μl) was prepared with DNA template (10 ng), KAPA SYBR FAST Master Mix (5 μl) (KAPA SYBR FAST kit; Kapabiosystems^©^), and each primer set (0.2 μM) in an ECO™ Real-Time PCR System Thermocycler (Illumina®). PCR amplification was performed at 95 °C for 2 min, and 45 cycles: 95 °C for 5 s, 61 °C for 15 s and 72 °C for 3 s.

### IL7R expression by RT-qPCR

Blood samples were centrifuged at 2000 rpm for 5 min, and the RNA was extracted in 800 μl of the precipitate with TRIzol™ reagent (Invitrogen, Thermo Fisher Scientific), according to the manufacturer instructions. Extracted RNA was treated with TURBO DNA-free™ kit (Ambion^®^, Life Technologies) and its concentration was determined by spectrophotometry (Epoch^®^, Biotek Instruments). Relative gene expression was determined by real time RT-PCR assay based on SYBR-Green detection. *TUBB* was used as gene for normalization^17^. cDNAs were obtained from 2 µg RNA using M-MLV Reverse Transcriptase (Promega^®^) and random primers (Promega^®^), according to the manufacturer’s guidelines. Primers to *IL7R* mRNA were designed using the Genbank sequence NM_002185.4 and the software Beacon Designer 8.02 (PREMIER Biosoft^®^) (F: 5’-CAGCAATGTATGAGATTA-3’; R: 5’-ATGGTTAGTAAGATAGGAT-3’). The PCR reaction was performed using the KAPA SYBR^©^ Fast qPCR (Kapa Biosystems^®^) with cDNA (2 µl) in a final volume (10 µl), according to the Eco™ Real-Time PCR system (Illumina^®^) protocol. PCR amplification was performed at 95 °C for 3 min, and 45 cycles: 95 °C for 3 s, 53 °C for 15 s and 72 °C for 3 s followed by the melting curve (55–95 °C). Gene expression fold changes (FC) were determined using the 2^−ΔΔCT^method^18^. The group used as control is indicated in each analysis.

### Quantification of membrane bound IL7Rα (CD127) expression on T cells and of peripheral T lymphocytes by flow cytometry

Blood samples were collected in EDTA tubes. Peripheral blood leukocytes were isolated by centrifugation (Boeco® Centrífuge) and stained with BD Horizon Brilliant Stain Buffer (563794 BD Pharmigen™) and BD Pharmigen Stain Buffer (FBS) (554656 BD Pharmigen™). Cells were incubated with specific antibodies, and later with 1× BD FACS Lysing Solution (641776 BD Pharmigen™). For the surface staining of peripheral blood mononuclear cells (1 × 10^6^), the followings antibodies of BD Pharmigen™ were used: APC mouse anti-human CD3 (555342), PerCPCy5.5 mouse anti-human CD4 (341654), FITC mouse anti-human CD8 (555634) and BV421 mouse anti-human CD127 (562436). Isotype-matched controls were used in all experiments: APC mouse IgG2a (555576), PerCPCy5.5 mouse IgG1 (550795), FITC mouse IgG1 (555748) and BV421 mouse IgG1 (562438). Dead cells were excluded by BD Horizon™ Fixable Viability Stain 780 (565388). Samples were acquired on the FACSCANTO™ II (BD Biosciences©). The data were analyzed using the FlowJo10.4® software.

### Quantification of soluble IL7R (sIL7R) by ELISA

Soluble IL7Rα in plasma was measured using Human IL-7 alpha ELISA (RayBiotech®) following manufacturer's instruction.

### Quantification of cytokines by Luminex^©^ technology

Plasma IL-7 was quantified by HCYTOMAG-60K kit (Milliplex MAP human cytokine/chemokine magnetic bead panel, MERCK®). The assay was run on MAGPIX® (Luminex©) with xPONENT software. Data were analyzed using a five-parameter logistic curve with Milliplex analyst software.

### Statistical analysis

Continuous variables were described as median and interquartile range (IQR) and categorical variables as frequency and percentage. Differences between groups were performed using the Chi square test for categorical data, and Student´s t and Mann–Whitney test for continuous variables, as appropriate. Parameters were compared according to predictor variables age, sex, and comorbidities. Days of illness were also analyzed in some parameters—as indicated in “Results”—because the immune response is dynamic over time. The area under the receiver operating characteristic curve (AUC) for predicting 30-day mortality and admission to ICU was calculated for plasmatic sIL7Rα. The association for each SNP of the *IL7R* with severity score and clinical outcomes (supplemental oxygen therapy, days of illness, ICU admission and death) in adults with CAP was evaluated through logistic regression model: crude (model 1), adjusted by age and sex (model 2) and adjusted by age, sex, and severity score (model 3). Odds ratios (ORs) and respective 95% confidence interval (CI) were reported as measure of associations. Hardy–Weinberg equilibrium of each SNP and association studies were tested using Armitage’s trend test (http://ihg.gsf.de/cgi-bin/hw/hwa1.pl). Alleles, genotypes and haplotype analysis were carried out with the Unphased 3.1.5 software^19^, as previously described^19^. Statistical significance was set at P value < 0.05. Data were analyzed using Stata 16.0, GraphPrism 8.4.2, and SPSS V25.0 software.

### Ethics approval and consent to participate

The study was approved by the Ethics committees at the Clínico Universidad de Chile Hospital (Act N12), the Universidad de Chile Facultad de Medicina (Act N009), and the South Metropolitan Health Service (Act N 142/2017). Written informed consent was obtained from each participant.

## Results

### Characteristics of studied population

Table 1 shows the general characteristics of 202 patients hospitalized with CAP. The clinical outcome was mechanical ventilation in 41 (20.3%) cases, and shock in 22 (10.9%) patients. Severe cases and comorbidities were more frequent in older patients (≥ 65 years) than in younger patients (p ≤ 0.02). The characteristics of the 100 women and 102 men were similar, except for higher frequency of asthmatics and mild cases (by PSI) in female than in male patients (13% vs 5%, p = 0.04; 30% vs 18%, p = 0.04) (see Supplementary Table 1 online).

**Table 1:** Clinical and epidemiological characteristics of 202 adults with CAP.

Characteristics	All patients	< 65 years	≥ 65 years	P
**Demographic**				
No, (%)	202 (100%)	64 (32%)	138 (68%)	–
Age, years: median [IQR]	73 [61–84]	54 [48–60]	79 [72–86]	< 0.001
Sex: Female Male	100/202 (49%) 102/202 (51%)	28/64 (44%) 36/64 (56%)	72/138 (52%) 66/138 (48%)	0.26
Days of illness: median [IQR]	5 [3–7]	6 [4–7]	5 [3–7]	0.02
< 7 days of illness	123/200 (62%)	32/64 (50%)	91/136 (67%)	0.02
**Comorbidity**				
Any comorbidity	162/200 (81%)	44/63 (70%)	118/137 (86%)	0.006
Hypertension	84/199 (42%)	16/62 (26%)	68/137 (50%)	0.002
Diabetes mellitus	64/201 (32%)	21/64 (33%)	43/137 (32%)	0.84
Asthma	18/202 (9%)	7/64 (11%)	11/138 (8%)	0.49
Chronic obstructive respiratory disease	51/202 (25%)	13/64 (20%)	38/138 (28%)	0.27
Cardiovascular disease	37/202 (18%)	6/64 (9%)	31/138 (22%)	0.03
Chronic liver disease	7/202 (3%)	0/64 (0%)	7/138 (5%)	0.07
Chronic kidney disease	18/201 (9%)	6/64 (9%)	12/137 (9%)	0.89
Neoplasia	12/201 (6%)	5/63 (8%)	7/138 (5%)	0.43
Neurological disease	34/200 (17%)	4/63 (6%)	30/137 (22%)	0.006
Sepsis	54/202 (27%)	12/64 (19%)	38/138 (28%)	0.2
**Pathogens**				
With agent detected	123/202 (61%)	37/64 (58%)	86/138 (62%)	0.54
Virus	68/123 (55%)	19/37 (51%)	49/86 (57%)	0.56
Bacteria	32/123 (26%)	11/37 (30%)	21/86 (24%)	0.54
Mixed (virus + bacteria)	23/123 (19%)	7/37 (19%)	16/86 (19%)	0.97
**Severity**				
*PSI*				
I y II (mild)	48/202 (24%)	35/64 (55%)	13/138 (9%)	< 0.001
III (moderate)	44/202 (22%)	11/64 (17%)	33/138 (24%)	0.28
IV y V (severe)	110/202 (54%)	18/64 (28%)	92/138 (67%)	< 0.001
*CURB-65*				
I (mild)	77/202 (38%)	51/64 (80%)	26/138 (19%)	< 0.001
II (moderate)	57/202 (28%)	8/64 (12%)	49/138 (35%)	< 0.001
III (severe)	68/202 (34%)	5/64 (8%)	63/138 (46%)	< 0.001
**Evolution**				
Supplemental oxygen therapy	117/202 (58%)	30/64 (47%)	87/138 (63%)	0.03
ICU admission	45/202 (22%)	21/64 (33%)	24/138 (17%)	0.01
Died up to 30 days	32/197 (16%)	2/62 (3%)	30/135 (22%)	< 0.001

The data are presented as n/N (%), unless otherwise specified. P values were calculated by Mann–Whitney test for continuous variables and by Chi-squared test for qualitative variables.

Respiratory pathogens were detected in 123 (61%) of 202 cases (Table 1). Viruses most frequently detected among 91 patients with viral detection were: influenza (37/91 cases; 40.7%) and rhinovirus (27 cases, 29.7%), and *S.pneumoniae* was the most frequently detected bacteria, in 42/55 cases (76.4%).

The median age of 22 asymptomatic adults without respiratory infection was 54.0 years (IQR: 43–63); 7 (36.8%) were men; 3 (15.8%) have co-morbidities (one type 2 Diabetes Mellitus, one asthma and one hypothyroidism); one was smoker (4.8%) and no one had alcohol consumption > 80 g/day.

### *IL7Rα* genetic variants

#### Allelic and genotypic frequencies

Several SNPs in the *IL7Rα* gene have been associated with immune and infectious diseases. In this study, three SNPS—rs6897932 (c.731-C > T), rs987106 (c.801–21-A > T) and rs3194051 (c.1066-A > G)—and their association with the severity of CAP were explored. Carriers of the rs987106TT and the rs3194051AG genotypes were more frequent in patients hospitalized in ICU (Table 2). The G allele in this last SNP is a risk allele according to Armitage’s test for association studies, with an odds ratio of 2.97 (p = 0.0003) between ICU-no ICU groups and of 2.52 with respect to the A allele between severe/mild cases according to PSI (p = 0.004). In addition, the rs3194051GG genotype was also more frequent in non-survivors than in survivor patients (Table 2). No differences were identified in the SNP rs6897932, except a higher number of carriers of the C allele and CC genotype among severe than in mild patients according to CURB-65. On the other hand, carriers of the rs3194051 AA genotype were significantly more frequent in patients who did not were admitted to the ICU (Table 2).

**Table 2:** Allelic and genotypic analysis of the SNP rs6897932, rs987106 and rs3194051 of the *IL7R*α gene in 202 adults with CAP.

Outcome		Supplemental oxygen therapy		ICU admission		Died up to 30 days		Severity by PSI		Severity by CURB-65	
		No	Yes	No	Yes	No	Yes	Mild	Severe	Mild	Severe
	*N*	85	117	157	45	165	32	48	110	77	68
**rs6897932**											
Allele (%)	C	65.9	69.2	67.5	68.9	66.7	73.4	64.6	69.1	63.6^a^	75.7
	T	34.1	30.8	32.5	31.1	33,3	26.6	35.4	30.9	36.4	24.3
Genotype (%)	CC	48.2	49.6	48.4	51.1	47.3	59.4	50.0	51.8	42.9^a^	60.3
	CT	35.3	39.3	38.2	35.6	38.8	28.1	29.2	34.6	41.6	30.9
	TT	16.5	11.1	13.4	13.3	13.9	12.5	20.8	13.6	15.6	8.8
**rs987106**											
Allele (%)	A	52.9	49.6	54.1^a^	40.0	51.2	50.0	53.1	48.2	49.4	55.2
	T	47.1	50.4	45.9	60.0	48.8	50.0	46.9	51.8	50.6	44.8
Genotype (%)	AA	30.6	24.8	29.3	20.0	26.7	34.4	35.4	26.4	24.7	35.3
	AT	44.7	49.6	49.7	40.0	49.1	31.3	35.4	43.6	49.4	39.7
	TT	24.7	25.6	21.0^b^	40.0	24.2	34.4	29.2	30.0	26.0	25.0
**rs3194051**											
Allele (%)	A	87.1	81.2	87.3^c^	71.1	84.6	79.7	88.5	80.0	85.7	80.9
	G	12.9	18.8	12.7	28.9	15.4	20.3	11.5	20.0	14.3	19.1
Genotype (%)	AA	75.3	66.7	75.8^b^	51.1	70.9	68.8	77.1	64.6	71.4	67.7
	AG	23.5	29.1	22.9^a^	40.0	27.3	21.9	22.9	30.9	28.6	26.5
	GG	1.2	4.3	1.3	8.9	1.8^a^	9.4	0.0	4.6	0.0^a^	5.9

Frequencies are shown as percentages. P values were calculated using the likelihood-ratio Chi-squared test for allelic or genotypic frequencies of each SNP in each group.

^a^p value < 0.05.

^b^p value < 0.01.

^c^p value < 0.001.

Multivariable logistic regression models were performed to assess if the genotype GG of rs3194051 was associated with the severity of illness compared to the other genotypes (GA, AA, or any of them (AA/GA)). rs3194051GG had a significant positive relationship with admission to ICU even in models adjusted by age, sex, and severity (Table 3). rs3194051GG was also positively associated with dead in relation to any of other genotypes in adjusted models by age and sex, but it did not reach statistical significance when severity was included in the model. These analyzes were not possible with the PSI score because of the small sample size.

**Table 3:** Logistic regression analysis of the SNP rs3194051 of the *IL7Rα* gene with severity and outcome in CAP adult.

	Severity by CURB-65	Supplemental oxygen therapy	Days of illness	ICU admission	Died up to 30 days
**GG versus GA**					
Model 1	4.00 (0.67; 23.94)	2.94 (0.32; 27.00)	1.14 (0.94; 1.39)	4.00 (0.67;23.94)	6.57 (1.10; 39.24)*
Model 2	5.84 (0.82; 41.47)	3.35 (0.36; 31.70)	1.14 (0.93; 1.38)	8.53 (0.97; 75.00)	10.72 (1.25; 91.88)*
Model 3	**–**	1.46 (0.11; 19.79)	1.24 (0.99; 1.54)	5.98 (0.61; 58.62)	6.23 (0.60;64.29)
**GG versus AA**					
Model 1	4.17 (0.74; 23.62)	4.10 (0.47; 36.02)	1.14 (0.93; 1.39)	10.35 (1.79; 59.85)*	5.32 (1.01; 28.08)*
Model 2	6.99 (0.84; 57.89)	4.46 (0.48; 41.56)	1.13 (0.91; 1.37)	10.65 (1.81; 62.50)*	10.96 (1.19; 101.14)*
Model 3	–	2.70 (0.24; 30.12)	1.23 (0.98; 1.53)	10.12 (1.66; 61.66)*	6.57 (0.70; 61.34)
**GG versus AA/GA**					
Model 1	4.13 (0.7; 23. 12)	3.75 (0.43; 32.70)	1.15 (0.95; 1.41)	7.56 (1.34; 42.73)*	5.62 (1.08; 29.22)*
Model 2	6.11 (0.87; 43.20)	3.90 (0.44; 34.75)	1.14 (0.94; 1.39)	8.97 (1.50; 53.45)*	10.46 (1.27; 86.07)*
Model 3	–	2.16 (0.20; 23.70)	1.24 (1.00; 1.54)*	7.14 (1.17; 43.37)*	6.14 (0.71; 52.85)

Numbers represent Odds Ratios and (95% Confidence intervals).

Model 1: Crude.

Model 2: Model 1 + age and sex.

Model 3: Model 2 + severity by CURB-65.

*p < 0.05.

According to age and sex, carriers of the C allele and CC genotype in the rs6897932 and the A allele in the rs987106 were significantly more frequent in older than in younger patients. This difference remained significant among men, but not among women (see Supplementary Table 2 online). In addition, the rs6897932CC and rs987106AA genotypes were more frequent in females than in males, but only in < 65 years patients. No differences were detected in the SNP rs3194051.

### *IL7R* haplotypes

The association between the haplotypes in the three SNPs of the *IL7Rα* gene and CAP severity was studied. The CAA allelic combination (rs6897932—rs987106—rs3194051) was less frequent in patients admitted to the ICU; the CTA haplotype was associated with dead and the CTG haplotype with admission at ICU (Table 4). The TTA haplotype was associated with mild illness, but only when CURB-65 score is applied.

Since age and comorbidities are factors associated to severity also were analyzed. Certain haplotypes were associated with any comorbidities and CAA allelic combination was more frequent in adults over 65 years (Table 4).

**Table 4:** Haplotypes of SNP rs6897932, rs987106 and rs3194051 of the IL7Rα gene in 202 adults with CAP.

SNPs					
rs6897932	rs987106	rs3194051	Groups	Frequency^a^	p value^b^
**Alleles**					
C	A	A	≥ 65 years	54.4% (150/276)	0.04
			< 65 years	43.8% (56/128)	
			ICU admission (yes)	40.0% (36/90)	0.01
			ICU admission (no)	54.1% (170/314)	
			Chronic liver disease (yes)	78.6% (11/14)	0.03
			Chronic liver disease (no)	50.0% (195/390)	
			Male ≥ 65 years	54.6% (72/132)	0.01
			Male < 65 years	37.5% (27/72)	
C	T	A	Non-survivors	3.1% (2/64)	0.001
			Survivors	0% (0/330)	
			Cardiac insufficiency (yes)	2.7% (2/74)	0.002
			Cardiac insufficiency (no)	0% (0/330)	
			Neurologic disease (yes)	2.9% (2/68)	0.001
			Neurologic disease (no	0% (0/332)	
C	T	G	ICU admission (yes)	28.9% (26/90)	< 0.001
			ICU admission (no)	12.7% (40/314)	
			Neurologic disease (yes)	25.0% (17/68)	0.03
			Neurologic disease (no)	14.8% (49/332)	
T	T	A	< 65 years	42.2% (54/128)	0.003
			≥ 65 years	27.5% (76/276)	
			Mild by CURB-65	36.4% (56/154)	0.02
			Severe by CURB-65	24.3% (33/136)	
			Male < 65 years	48.6% (35/72)	0.002
			Male ≥ 65 years	27.3% (36/132)	

^a^(n haplotype/n total haplotypes).

^b^P values were calculated using the likelihood-ratio Chi-squared test for allelic or genotypic frequencies of each SNP in each group.

Analysis of the genotypes of all the SNPs studied showed that the CC-TT-AG combination was significantly more frequent in severe cases than in mild cases, and the CC-TT-GG genotype in the severe by CURB-65 and non-survivors patients (Table 5). So, G allele in the SNP rs3194051 is associated with severity of the CAP in adults.

**Table 5:** Genotypes of SNP rs6897932, rs987106 and rs3194051 of the IL7Rα gene in 202 adults with CAP.

SNPs					
rs6897932	rs987106	rs3194051	Groups	Frequency^a^	p value^b^
**Genotypes**					
C/C	A/A	A/A	Male ≥ 65 years	31.8% (21/66)	0.02
			Male < 65 years	11.1% (4/36)	
			Female < 65 years	32.1% (9/28)	0.03
			Male < 65 years	11.1% (4/36)	
C/C	A/T	A/G	< 7 days of illness	22.0% (27/123)	0.03
			≥ 7 days of illness	10.4 (8/77)	
			Neurologic disease (yes)	32.4% (11/34)	0.02
			Neurologic disease (no)	15.7% (26/166)	
C/C	T/T	G/G	Severe by CURB-65	5.9% (4/68)	0.03
			Mild by CURB-65	0% (0/77)	
			ICU admission (yes)	8.9% (4/45)	0.008
			ICU admission (no)	1.3% (2/157)	
			Non-survivors	9.4% (3/32)	0.02
			Survivors	1.8% (3/165)	
C/T	T/T	A/G	ICU admission (yes)	17.8% (8/45)	0.01
			ICU admission (no)	5.7% (9/157)	
			Male < 65 years	13.9% (5/36)	0.03
			Male ≥ 65 years	3.0% (2/66)	
T/T	T/T	A/A	< 65 years	20.3% (13/64)	0.04
			≥ 65 years	10.1% (14/138)	

^a^(n haplotype/n total haplotypes).

^b^P values were calculated using the likelihood-ratio Chi-squared test for allelic or genotypic frequencies of each SNP in each group.

### *IL7Rα* gene expression

*IL7Rα* gene expression was lower in 13 non-survivors adults than in 112 survivors with CAP (FC: 0.26 vs 1.0; p = 0.01), but similar between 22 patients in ICU and 107 cases without admission to ICU (p = 0.6). According to severity of CAP defined by clinical scores, gene expression was lower in 57 severe cases than in 39 mild cases by PSI (FC: 0.27; p = 0.009), but similar between cases classified by CURB-65.

In relation to genotypes of the SNPs studied, expression of *IL7Rα* gene was significantly lower in 58 carriers of the CT genotype than in 54 carriers of the CC genotype of the rs6897932 (FC: 0.28; p = 0.01). Although differences were not statistically significant, expression was lower in 66 carriers of the AT and 28 of the TT genotypes than in 35 carriers of the AA genotype in the rs987106 (FC: 0.33 and 0.25, respectively) (p > 0.1) and higher in 30 carriers of the AG genotype than in 97 carriers of the AA genotype in the rs3194051 (FC: 1.79; p = 0.5).

*IL7Rα* gene expression was lower in 81 adults over 65 years old with CAP than in 48 adults < 65 years (FC: 0.55 vs 1.0, respectively), and was higher in 62 men than in 67 women (FC: 2.31 vs 1.0, respectively), although a statistical significance was not gotten. No differences were found comparing days of illness or pathogen detected.

### Proportion of CD3^+^ lymphocytes

**Figure 1:**
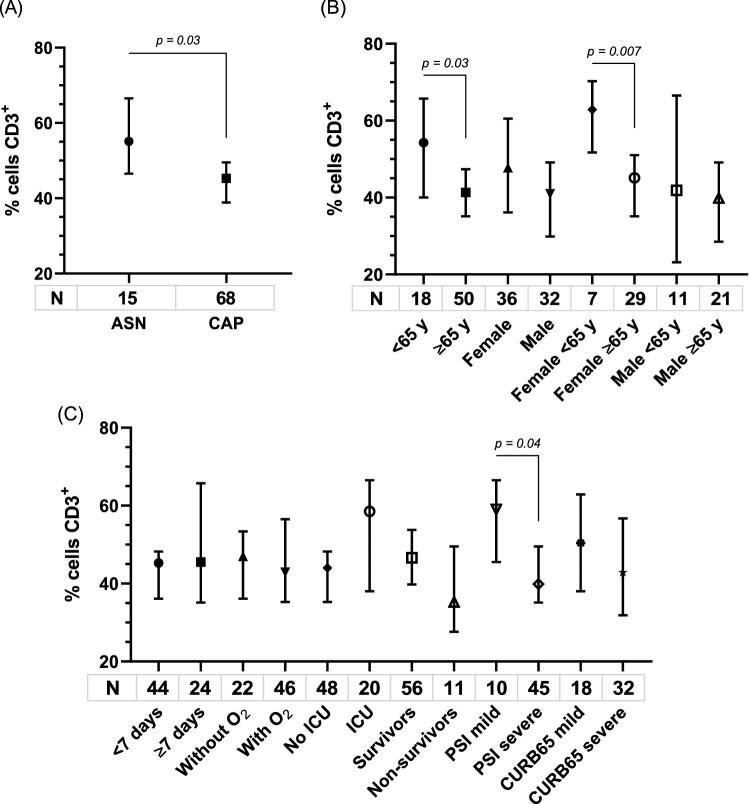
Systemic CD3^+^ lymphocytes in CAP and asymptomatic adults (ASN) (**A**), according to age, sex (**B**), and outcome (**C**). Data are shown as median and 95% CI. P values were obtained by Mann–Whitney test.

Given the connection between IL7R axis and T lymphocytes, these subpopulations were studied in 68 CAP and in 15 asymptomatic adults by flow cytometry. Proportion of the CD3^+^ lymphocytes was significantly lower in patients (median: 45.3% vs 55.1% [IQR: 31.8–59.58 and 46.5–66.5]) (Fig. 1A), and in adults ≥ 65 years old than in < 65 years old (median: 41.4% vs 54.3% [IQR: 30.5–54.5 and 39.9–65.9]) (p = 0.03), difference that remained statistically significant only in women (median: 45.1% vs 62.9% [IQR: 31.8–60.6 and 53.4–66.5]( (p = 0.007) (Fig. 1B). Also CD3^+^TL was lower in non-survivors patients than in survivors (median: 35.3% vs 46.6% [IQR: 29.9–48.2 and 35.2–60.8; p = 0.1), but statistical significance was not reached. CD3^+^ lymphocytes proportion was lower in severe patients than in mild cases according to PSI (median: 39.9% vs 59.0% [IQR: 30.3–58.6 and 50.1–66.5]; p = 0.04) (Fig. 1C), and similar by CURB-65 (median: 49.9% vs 50.4% [IQR: 30.4–59.6 and 36.0–63; p = 0.2).

### Expression of membrane-bound IL7Rα (CD127) on T lymphocytes

**Figure 2:**
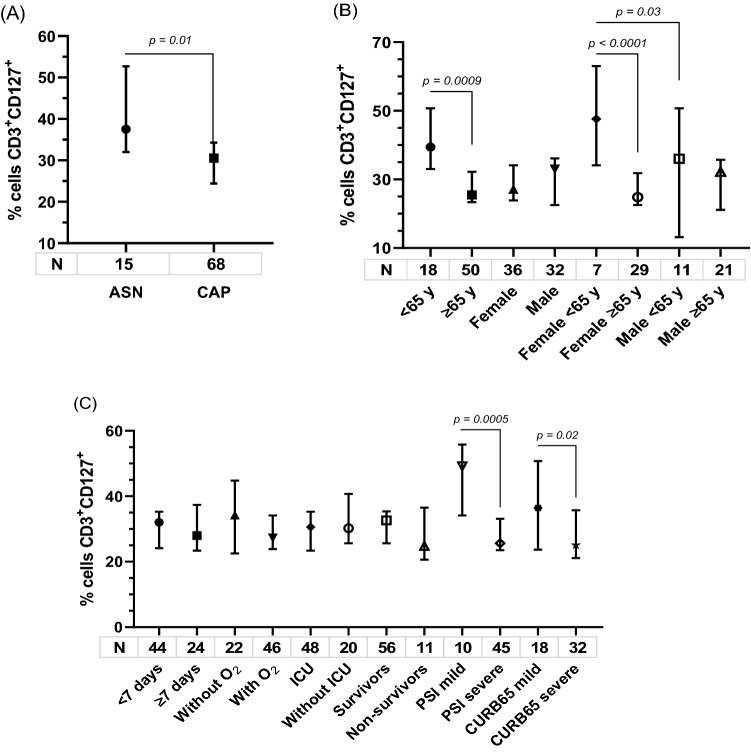
Systemic CD3^+^CD127^+^ lymphocytes in CAP and asymptomatic adults (ASN) (**A**), according to age, sex (**B**) and outcome (**C**). Data are shown as median and 95% CI. P values were calculated by Mann–Whitney test.

T lymphocytes expressing α-receptor membrane-bound IL7Rα (CD127) were studied by flow cytometry. Significantly lower proportion of the CD3^+^CD127^+^ lymphocytes was recorded in patients as compared to asymptomatic adults (median: 30.5% vs 37.5% % [IQR: 22.4–37.8 and 32.0–52.7]; p = 0.01) (Fig. 2A). Similarly, the proportion was significantly lower in severe patients than in mild cases classified by both PSI (median: 25.6% vs 49.1% [IQR: 21.7–35.3 and 37.1–54.7]) and CURB-65 (25.0% vs 36.4% [IQR: 18.3–36.4 and 23.4–51.5]) (p ≤ 0.02). Although statistical significance was not reached, CD3^+^CD127^+^ lymphocytes also were lower in non-survivors patients than in the survivors (median: 24.8% vs 32.6% % [IQR: 21.0–32.3 and 22.5–40.6]; p = 0.2) (Fig. 2B).

Significantly lower proportion of the CD3^+^CD127^+^ lymphocytes was recorded in older than in younger patients (median: 25.4% vs 39.4% [IQR: 21.1–34.9 and 31.4–51.5]; p = 0.0009), being also significant among women (median: 24.8% vs 47.6% [IQR: 20.0–32.2 and 40.7–55.8]; p < 0.0001) (Fig. 2C).

**Figure 3:**
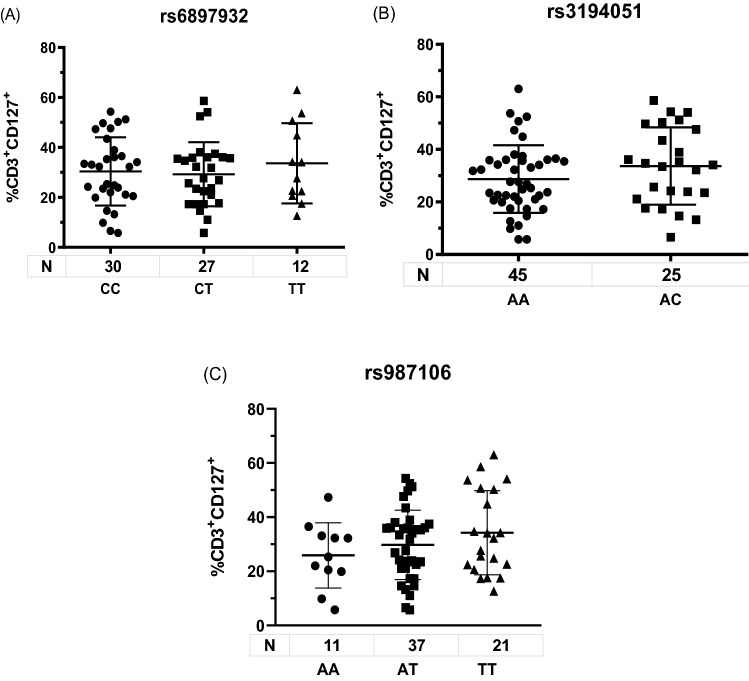
CD3^+^CD127^+^ lymphocytes in blood from adults with CAP according to SNP. Data are shown as median and 95% CI. p > 0.1, by t test.

There were no significant differences according to genotypes (Fig. 3A–C) (rs3194051GG was excluded because only one patient could be analyzed) nor pathogen detected: 40 viral cases (median: 32.0% [IQR: 23.6–37.2]) and 62 patients with bacterial detection (24.6% [IQR: 16.2–39.7]) (p = 0.4).

The mean fluorescence intensity of CD3^+^CD127^+^ lymphocytes was determined as an estimate of the number of receptors per cell, being significantly higher in the 11 non-survivors patients than in the 56 survivors (median: 5599 vs 3377 [IQR: 3741–7076 and 2624–4482]; p = 0.001). No differences were detected between 15 patients in ICU and 48 without admission to ICU (median: 3269 vs 3688 [IQR: 2401–3838 and 2918–5594]; p = 0.1); between 45 severe patients and 10 mild cases according to PSI (median: 3563 vs 3085 [IQR: 2890–4848 and 2371–4212]; p = 0.2); between 32 severe patients and 36 mild cases according to CURB-65 (median: 3593 vs 3367 [IQR: 2886–5355 and 2684–4736]; p = 0.3) and between 18 older adults and 50 younger (median: 3369 vs 3557 [IQR: 2565–4695 and 2892–4874]; p = 0.5).

**Figure 4:**
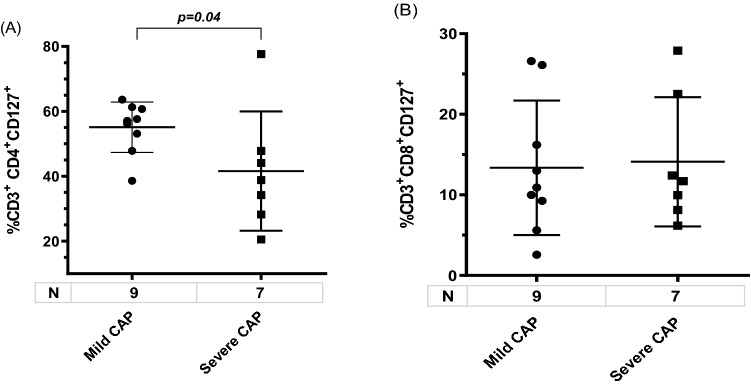
Systemic CD4^+^CD127^+^ (**A**) and CD8^+^CD127^+^ (**B**) T lymphocytes in CAP adult according to severity by PSI. Data are shown as median and 95% CI. P values were obtained by t test.

Although expression of membrane bound IL7Rα (CD127) in CD4^+^ and CD8^+^ T cells was quantified in few patients, a significant lower proportion of CD4^+^CD127^+^ cells, but no of CD8^+^CD127^+^ cells, were detected in severe cases as compared to mild cases classified by PSI (Fig. 4A,B).

### Quantification of soluble IL7R

**Figure 5:**
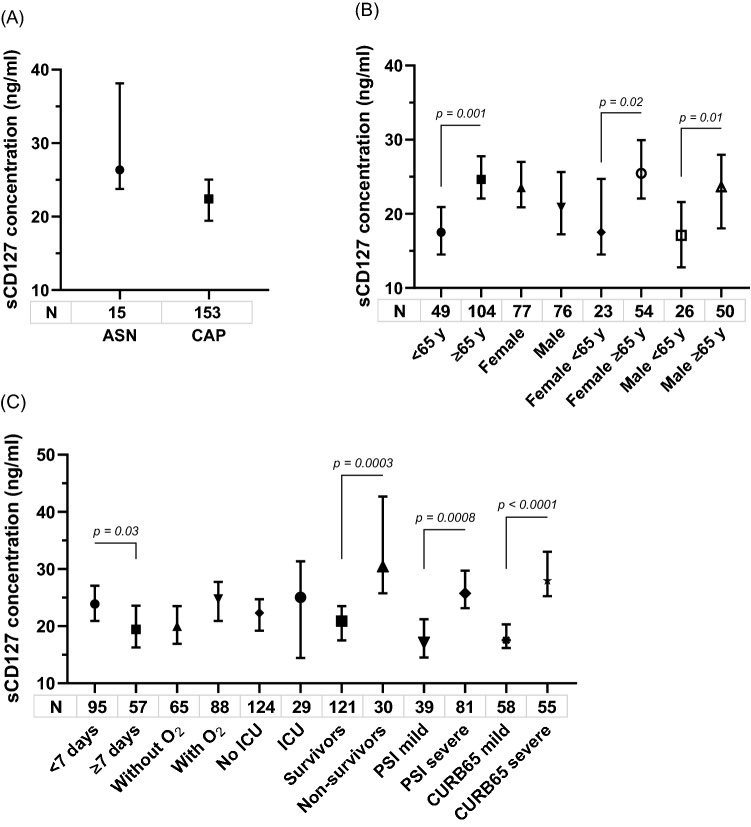
Plasmatic soluble IL7R from asymptomatic and CAP adults (**A**), according to age, sex (**B**) and outcome (**C**). Data are shown as median and 95% CI. P values were calculated by Mann–Whitney test.

Soluble IL7R (sIL7R) is associated with the homeostasis of the IL-7/IL7R axis. In this study, plasmatic levels of sIL7Rα were higher in asymptomatic adults than in adults with CAP, although statistical significance was not developed (median: 26.3 vs 22.4 ng/ml [IQR: 23.8–38.1 and 15.2–33.4; p = 0.09) (Fig. 5A). Also levels of sIL7R were significantly higher in adults ≥ 65 years than in patients < 65 years (median: 24.6 vs 17.5 [IQR: 16.9–35.0 and 11.8–25.4]; p = 0.001) (Fig. 5B). The non-survivors patients showed significantly higher sIL7R concentrations than the survivors (median: 30.4 vs 20.9 ng/ml [IQR: 21.6–46.4 and 14.4–29.3]; p = 0.0003). The same happened in the severe cases with respect to mild cases according to PSI (median: 25.7 vs 17.1 [IQR: 16.2–38.2 and 12.8–22.5]; p = 0.0008) and CURB-65 (median: 27.9 vs 17.5 [IQR: 21.2–40.4 and 13.4–24.5]; p < 0.0001) (Fig. 5C). sIL7R also was higher in the 29 patients with mechanical ventilation than in 124 without MV, but the difference was not significant (median: 29.4 ng/ml vs 21.39 ng/ml; p = 0.05).

**Figure 6:**
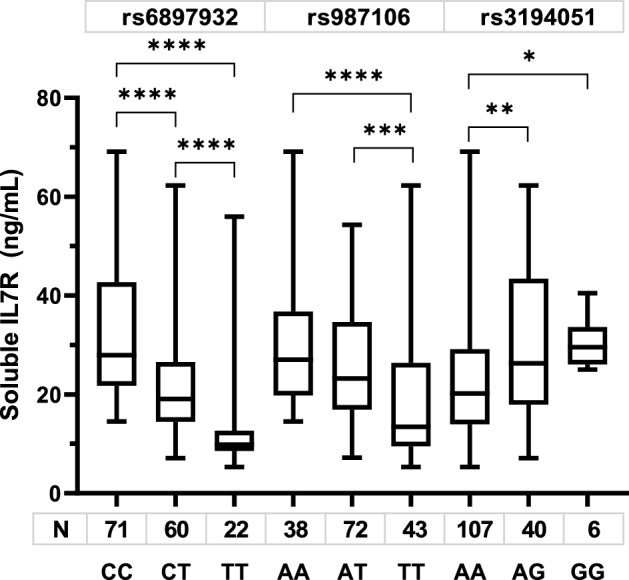
Soluble IL7R according to the genotypes of the three SNPs in adult CAP. Median, 25–75% percentile, minimum and maximum are indicated. P values were obtained by Mann–Whitney test, (*) p < 0.05, (**) p < 0.01, (***) p < 0.001, (****) p < 0.0001.

Significant differences in levels of sIL7R were detected in the patients according to the genotypes of the three SNP studied. Thus, the highest levels were detected in carriers of rs6897932CC, rs987106AA, and rs3194051AA genotypes; while the lowest concentrations were observed in adults with rs987106TT, rs3194051AA genotypes, and the lowest of all, the rs6897932TT carriers (Fig. 6).

An evaluation of the predictive capacity of the plasmatic sIL7R levels was performed by an area-under-the curve (AUC) analysis. AUC was 0.71 (SE 0.05; 95% CI: 0.60–0.81) for prediction of 30-day mortality and 0.48 (SE 0.06; 95% CI: 0.36–0.60) for admission at ICU. Regarding sIL7R values, the best threshold calculated on the basis of minimum d (minimum distance between ROC curve and point of sensitivity and specificity = 1) was 25.04 ng/ml. For this cutoff value, sensitivity was 73.3%, specificity was 66.1%, 67.6% was classified correctly, LR ( +) was 2.1642 and LR (−) was 0.4033. Among 113 CAP adults with sIL7R levels above 25.04 ng/ml, 85 (75.2%) were ≥ 65 years, 30 (26.5%) were admitted to ICU, 75 (66.4%) were classified as severe by PSI and 50 (44.2%) by CURB-65 and, whereas only 53 (59.6%), 8 (8.98%), 35 (39.3%) and 18 (20.2%) were in the 89 cases with levels below this cut-off value, respectively (p < 0.02).

Moreover, to determine whether plasma concentrations of the sIL7R correlate with the level of membrane-bound receptor expression on T cells (CD3^+^CD127^+^ lymphocytes), ratio of membrane to soluble receptor was evaluated, being significantly lower in severe than in mild cases by both PSI and CURB-65, and in adults ≥ 65 years than in adults < 65 years. Although not statistically significant, ratio was also lower in non-survivors than in survivor patients and in cases in ICU than in without admission to ICU (Table 6).

**Table 6:** Ratio of the membrane-bound receptor expression on T cells to the soluble receptor plasma IL7Rα in adults with CAP.

Parameter	n	Median	IQR	p
**By PSI**				
Severe	37	0.95	0.59–1.69	0.001
Mild	8	4.28	1.86–5.42	
**By CURB-65**				
Severe	30	0.85	0.53–1.63	0.02
Mild	22	1.51	0.80–2.63	
**ICU**				
Yes	7	0.84	0.47–1.77	0.6
No	42	1.10	0.59–2.04	
**Death up to 30 days**				
Yes	11	0.80	0.58–1.30	0.1
No	42	1.37	0.70–2.21	
**Age**				
< 65 years	14	2.38	1.27–4.73	0.001
≥ 65 years	40	0.92	0.53–1.59	

P values were calculated by Mann–Whitney test.

Although the linear regression model showed no relationship between sIL7R concentration and days of illness (coefficient −0.02; 95% CI: −0.06 to 0.014; p = 0.29), given sIL7R levels were significantly higher in 95 patients with less 7 days of illness than in 57 cases with > 7 days (median: 23.8 vs 19.4 ng/ml; p = 0.03), the analyses were repeated excluding the latter cases. Significant differences remained in all comparisons previously shown, except in higher levels of sIL7R between carriers of AG vs AA and GG vs AA genotypes in the rs3194051. On the contrary, the greatest levels of sIL7R in carriers of CC vs CT genotypes in the rs6897932 and AA vs AT genotypes in the rs987106 achieved statistical significance (see Supplementary Table 3 online).

### Concentration of IL-7

Since IL-7 activates its receptor and plays a transcendental role in T cell homeostasis, IL-7 levels were evaluated. Significantly higher levels of plasmatic cytokine IL-7 were detected in adults with CAP than in asymptomatic adults. Levels were lower in patients in ICU, with oxygen therapy and in the non-survivors patients, although the last difference was not statistically significant (Table 7). No significant differences were detected in IL-7 levels by gender, age, or severity according to both clinical scores (Table 7), or by genotypes either (see Supplementary Table 4 online).

**Table 7:** Plasmatic IL7 from CAP and asymptomatic adults according to severity, age, sex and outcome.

IL7 (pg/ml)	N	Median	IQR	P
**Adults**				
CAP Asymptomatic	169 22	5.0 2.8	2.7–7.5 1.8–4.6	0.008
**Severity by**				
PSI: severe Mild CURB-65: severe Mild	98 38 56 64	4.3 5.3 4.7 5.2	2.1–6.7 3.0–9.3 2.7–6.9 2.7–8.4	0.08 0.6
**ICU**				
Yes No	39 130	3.3 5.3	1.8–5.7 3.0–7.9	0.02
**Oxygen therapy**				
Yes No	133 66	4.3 5.3	2.4–7.1 3.0–8.6	0.03
**Death up to 30 days**				
Yes No	28 137	4.5 5.0	2.7–6.0 2.5–7.6	0. 3
**Sex**				
Women Men	80 89	4.3 5.0	2.4–7.1 2.8–8.3	0.3
**Age**				
≥ 65 years < 65 years	112 57	4.5 5.3	2.1–7.1 3.0–9.0	0.09
**Age and sex**				
Women ≥ 65 years < 65 years Man ≥ 65 years < 65 years	57 23 55 34	4.0 5.3 5.04 5.21	2.1–6.7 4.0–9.2 2.7–7.8 3.0–9.2	0.03 0.80

P values were calculated by Mann–Whitney test.

No correlation between plasmatic IL-7 and sIL7R concentrations was found in 123 CAP cases studied (Spearman’s rank correlation coefficients, r = −0.11; 95% IC: −0.2895 to 0.07025; p = 0.21); not with LTCD3^+^ (r = 0.11; 95% IC:−0.1433 to 0.3543; p = 0.3); CD127^+^ (r = 0.10; 95% IC: −0.1546 to 0.3576; p = 0.40), or age either (r = −0.0663; p = 0.3).

According to pathogen detected, IL-7 level was lower in 26 patients with bacteria than in 57 viral cases (median: 3.6 vs 5.3 pg/ml [IQR: 1.8–5.3 and 3.4–7.6]; p = 0.01), while both viral and bacterial cases were similar to 19 patients with mixed infections (median: 4.3 pg/ml [IRQ: 1.8–9.6]; p > 0.4).

## Discussion

To the best of our knowledge, this is the first study of the genic/protein expression IL-7/IL7R axis in adults with CAP, looking for a new potential severity factor. High plasmatic levels of soluble receptor IL-7 (sIL7R) and two IL7R gene polymorphisms studied were associated to CAP severity. Thus, the non-survivors patients had higher plasmatic levels of sIL7R than the recovered patients, as well as non-significant lower levels of both the genic expression of IL7R and IL-7, and of circulating CD3^+^TL and CD3^+^CD127^+^TL. These results agree with previous reports on the regulatory role of sIL7R in the capture of IL-7^6^. Its increase produces a diminishing of the circulating IL-7 level, which interacts with the lymphocyte’s membrane receptor, determining lymphopenia. The increase in membrane receptor levels could be due to attempts to compensate for the decrease in IL7. CD4^+^127^+^ more than CD8^+^127 T lymphocytes were affected, which is very important given the essential role of these cells in the immune response.

The significant relationship between high sIL7R levels and lethality risk in CAP has also been described in patients suffering sepsis, where cases with lower sIL7R have better outcomes, proposing its use as a risk biomarker^20^. Actually, in our study those cases with fatal sepsis had higher levels of sIL7R than those recovered (medians: 30.8 vs 19.7 ng/ml; p = 0.01) and the increased sIL7R levels persisted when the case severity was defined by other parameters, such as ICU admission, PSI and CURB-65 severity scores. AUC of sIL7R for fatality was similar to those Score index (AUC = 0.71), being similar to the obtained in septic cases (AUC: 0.774- 0.846). Furthermore, it is interesting to outstand that cases classified as moderated by PSI and CURB-65 had sIL7R levels similar to severe cases and also over the mild cases levels, (median: 22.41, 25.76 and 17.18 ng/ml, respectively), suggesting that those cases should have been managed as severe ones; some of them were after admitted to ICU or passed on.

In addition, two IL7R gene polymorphisms studied were associated to CAP severity. rs3194051GG genotype carriers displayed five times more lethal outcomes than cases with no GG. These patients also predominated among the cases admitted to UCI, as well as rs987106TT genotype carriers. GG genotype has been associated with autoimmune pathologies like type I diabetes^21^ and multiple sclerosis^11^, diseases that were not reported in the cases studied. Both SNPs are located in a potential regulatory zone whose effects have not been defined^14^ and which it is different to those related to mutations observed in the combined severe immunodeficiency, disease that is not present in the adults studied.

The different production of sIL7R is the more noticeable effect of polymorphisms^12,22^. In this study, changes in the sIL7R levels were detected in the three SNP according to the genotype. Thus, the plasmatic level was higher in rs3194051GG, rs987106AA and rs6897932CC genotypes, while the lowest was in the carriers of rs6897932TT. It is striking that the highest levels are detected in the genotype associated with severe and fatal cases, while the lowest levels are identified in the genotype TT, described previously as a protector for multiple sclerosis^11^, through of reduced exon splicing and production of soluble IL7R^23,24^.

Although the plasmatic levels of sIL7R were associated to genotypes, no association was observed between the genotypes and the expression of *IL7R* gene in blood. This last result should be interpreted with caution by two limitations of this study: first, the analyzed mRNA corresponded to the receptor attached to the membrane and not to the soluble one, and second because RNA is extracted from all blood cells, which does not detect changes in expression in a certain cell type. Association between genotypes and receptor expression in the LT membrane was also not detected. It would be interesting to explore whether this condition also occurs in other types of leukocytes such as monocytes. Recently, a key role of the latter cells has been raised in the biological pathway of diseases associated with cytokine IL-7 and its receptors^24^.

The highest proportion of severe cases and deaths detected among those over 65 is consistent with the risk of severity described in older adults. They also had higher levels of sIL7R and lower percentage of CD127^+^ T lymphocytes in relation with the younger cases, which agree with the role of IL-7/IL7R axis in the immunosenescense^6^. However, the *IL7R* expression was not significantly diminished in older adults as it has been published^25^, and further studies are necessary. On the other side, since sIL7R levels are increased over 65 years old and all the fatal cases were over that age, it could be argued that the high level was due to the age factor. However, among those over 65 years of age, sIL7R levels were significantly lower in recovered than in non-survivors cases (median: 23.3 vs 33.2 ng/ml; p = 0.004). Therefore, the old age would not be the only cause of the high level of the sIL7R.

There were no gender differences, but some parameters showed differences when they were analyzed according to age. Thus, while the proportion of CD3^+^ and CD127^+^ T lymphocytes in females was lower over 65 years old than in younger ages, the genotype rs987106CC in men was significantly more frequent in cases over 65 years old. There are no explanations for this fact or its influence in the CAP outcome.

Although the IL7/IL7R axis has been associated with certain autoimmune conditions like type 1 diabetes mellitus, rheumatoid arthritis and multiple sclerosis^26^, the more frequent comorbidities in patients over 65 years old does not explain the higher sIL7R concentration detected. They represent other type of pathologies, mostly cardiovascular and neurologic diseases, and no differences were observed in the levels of sIL7R between cases with and without these comorbidities. Therefore, the presence of comorbidities would not interfere with the level of sIL7R as severity biomarker in adult CAP. Furthermore, its determination can be quantitatively performed with a routine technique such as ELISA, which it can be easily implemented in clinical hospitals.

Plasmatic concentrations of IL-7 had no variation in relation to severity conditions described or with genotypes or sIL7R plasmatic levels, unlike that described in sepsis^20^. Probably, the SNP-associated mechanisms affecting IL-7 levels are more complex, and independent mechanisms of sIL7R regulate IL-7 levels^23^. This needs to be resolved before implementing recombinant human IL7 therapy as proposed given severe CAP-associated lymphopenia^27^.

There were no differences in sIL7R and IL-7 in relation to pathogens detected. Just a higher IL-7 level in viral versus bacterial CAP was detected, probably explained by the role of IL-7 in promoting antiviral activity of T lymphocytes^14^.

A limiting condition of the study was the patient evaluation just one time, which impedes to have a dynamic vision of quantified parameters. However, only differences in sIL7R were detected according to the time of evolution, being significantly lower in cases with over one week of evolution. No differences were seen in cases with more or less than 3 days of evolution, neither between 1 and 3 days as in sepsis^20^. Another limitation of the study could be the mixed analysis of patients with and without detected pathogen; however, the absence of differences in the demographic and clinical characteristics, in the evolution and in the parameters studied between the two groups (data not shown) suggest that the results would not be affected by the detection of pathogens.

The results in our study explore alterations in the IL-7/sIL7R/mIL7R axis in adult community-acquired pneumonia. The aforementioned axis formulated in relation to autoimmune diseases like multiple sclerosis, includes lower levels of sIL7R and augmented relationship of mIL7R/sIL7R^26^. For adult CAP with risk of severity we propose a pathogenic model characterized by a pattern of a high level of sIL7R and a diminished IL-7 concentration, which tend to decrease T lymphocyte activity and therefore, the control of the infection.

The development of new biomarkers of severity could improve therapy of CAP in adults. In this study, parameters associated with severity were identified which, although currently have limited clinical application, it is of interest to explore their usefulness in conjunction with other biomarkers, since the complexity of the pathogenesis of the neumonia, a panel of different biomarkers is probably required, including genetic and immunological parameters.

In conclusion, SNPs of IL7R would be related to the severity of adults with CAP, since unfavorable IL7R genotypes (rs987106 TT, and rs3194051GG) had a worse prognosis. Our results constitute the first description of a significant association between sIL7R levels and SNPs in rs3194051, rs6897932 and rs987106 in adults with CAP. The increase in plasma level of sIL7R can contribute to identify adults with CAP in risk of death. Their routine implementation and even automated quantification, by the ELISA technique can be easily executed in hospitals.

## Supplementary Information

Supplementary Tables.

## Data Availability

All of the datasets in the current study are available from the corresponding author on reasonable request.

## Abbreviations

CAP: Community acquired pneumonia
IL7Rα or CD127: Membrane-bound receptor interleukin 7
sIL7R: Soluble receptor interleukin 7
SNP: Single-nucleotide polymorphism
ICU: Intensive care
PSI: Pneumonia Severity Index
CURB-65: Confusion, uremia, respiratory frequency, blood pressure, 65 years old
IL-7: Interleukin 7
NK: Natural killer cells
SCID: Severe combined immunodeficiency
RNAm: Messenger ribonucleic acid
HCoV: Human coronavirus
DNA: Deoxyribonucleic acid
PCR-HRM: Polymerase chain reaction-high resolution melting
RT-qPCR: Real time reverse transcription-polymerase chain reaction
cDNA: Complementary deoxyribonucleic acid
ELISA: Enzyme-linked immunosorbent assay
FC: Fold changes
IQR: Interquartile range
AUC: Area under the receiver operating characteristic curve
ORs: Odds ratios
CI: Confidence interval
COPD: Chronic obstructive pulmonary disease
LR: Likelihood ratios
