# Supplementary material for: IL-7/IL7R axis dysfunction in adults with severe community-acquired pneumonia (CAP): a cross-sectional study
Source: Sci Rep. 2022 Jul 30;12:13145. doi: 10.1038/s41598-022-13063-x (PMC9339003; doi:10.1038/s41598-022-13063-x)
Supplement: Supplementary file 1 — Supplementary Tables. [file 41598_2022_13063_MOESM1_ESM.pdf]

Supplementary Table 1

Variables	Total (n:202)	Women (n:100)	Men (n:102)	p
**Demographic**				
Age (years): median [IQR]	73 [60 – 84]	74 [61 – 84]	70 [60 – 81]	0.21
< 65 years: n/N (%)	64/202 (32)	28/100 (28)	36/102 (35)	0.26
Days of illness: median (IQR)	5 (3 - 7)	5 (3 - 7)	5 (3 - 7)	0.56
< 7 days of illness: n/N (%)	123/200 (62)	61/100 (61)	62/100 (62)	0.88
**Comorbidity**				
Any comorbidity n/N (%)	162/200 (81)	82/99 (83)	80/101 (79)	0.51
Hypertension	84/199 (42)	44/99 (44)	40/100 (40)	0.53
Diabetes mellitus	64/201 (32)	28/99 (29)	36/102 (35)	0.29
Asthma	18/202 (9)	13/100 (13)	5/102 (5)	0.04
Chronic obstructive respiratory disease	51/202 (25)	27/100 (27)	24/102 (24)	0.57
Cardiac insufficiency	37/202 (18)	19/100 (19)	18/102 (18)	0.80
Chronic liver disease	7/202 (3)	4/100 (4)	3/102 (3)	0.68
Chronic kidney disease	18/201 (9)	7/99 (7)	11/102 (11)	0.36
Neoplasia	12/201 (6)	4/99 (4)	8/102 (8)	0.26
Neurologic disease	34/200 (17)	19/99 (19)	15/101 (15)	0.41
**Pathogens**				
With pathogen detected	123/202 (61)	65/100 (65)	58/102 (57)	0.24
Virus	68/123 (55)	36/65 (55)	32/58 (55)	0.98
Bacteria	32/123 (26)	18/65 (28)	14/58 (24)	0.65
Mixed	23/123 (19)	11/65 (17)	12/58 (21)	0.59
**Severity according to**				
PSI				
Group I y II (mild)	48/202 (24)	30/100 (30)	18/102 (18)	0.04
Group III (moderate)	44/202 (22)	22/100 (22)	22/102 (22)	0.94
Group IV y V (severe)	110/202 (54)	48/100 (48)	62/102 (61)	0.07
CURB-65				
Group I (mild)	77/202 (38)	35/100 (35)	42/102 (55)	0.37
Group II (moderate)	57/202 (28)	27/200 (7)	30/102 (29)	0.70
Group III (severe)	68/202 (34)	38/100 (38)	30/102 (29)	0.20
**Outcome**				
Supplemental oxygen therapy	117/202 (58)	59/100 (59)	58/102 (57)	0.76
ICU admission	45/202 (22)	25/100 (25)	20/102 (20)	0.36
Died up to 30 days	32/197 (16)	18/99 (18)	14/98 (14)	0.46

Clinical and epidemiological characteristics of adults with CAP according to sex. P values were calculated by Mann-Whitney test for continuous variables and by Chi-squared test for qualitative variables

Supplementary Table 2

	Age		Sex		Female		Male		Age <65 y		Age ≥ 65 y	
Group	< 65 y	≥ 65 y	F	M	< 65 y	≥ 65 y	< 65 y	≥ 65 y	F	M	F	M
n	64	138	100	102	28	72	36	66	28	36	72	66
**rs6897932**												
**Allele (%)**												
**C**	57.8[b]	72.5	70.5	65.2	66.1	72.2	51.4[b]	72.7	66.1	51.4	72.2	72.7
**T**	42.2	27.5	29.5	34.8	33.9	27.8	48.6	27.3	33.9	48.6	27.8	27.3
**Genotype (%)**												
**CC**	35.9[a]	55.1	51.0	47.1	50.0	51.0	25.0[c]	59.0	50.0[a]	25.0	51.0	59.0
**CT**	43.8	34.8	39.0	36.3	32.0	42.0	53.0[b]	27.0	32.0	53.0	42.0	27.0
**TT**	20.3[a]	10.1	10.0	16.7	18.0	6.9	22.0	14.0	18.0	22.0	6.9	14.0
**rs987106**												
**Allele (%)**												
**A**	43.8[a]	54.4	53.5	48.5	51.8	54.2	37.5[a]	54.6	51.8	37.5	54.2	54.6
**T**	56.2	45.6	46.5	51.5	48.2	45.8	62.5	45.4	48.2	62.5	45.8	45.4
**Genotype (%)**												
**AA**	20.3	30.4	30.0	24.5	32.0	29.0	11.0[a]	32.0	32.0[a]	11.0	29.0	32.0
**AT**	46.9	47.8	47.0	48.0	39.0	50.0	53.0	45.0	39.0	53.0	50.0	45.0
**TT**	32.8	21.7	23.0	27.5	29.0	21.0	36.0	23.0	29.0	36.0	21.0	23.0
**rs3194051**												
**Allele (%)**												
**A**	85.9	82.6	84.0	83.3	85.7	83.3	86.1	81.8	85.7	86.1	83.3	81.8
**G**	14.1	17.4	16.0	16.7	14.3	16.7	13.9	18.2	14.3	13.9	16.7	18.2
**Genotype (%)**												
**AA**	73.4	68.8	70.0	70.6	75.0	68.0	72.0	70.0	75.0	72.0	68.0	70.0
**AG**	25.0	27.5	28.0	25.5	21.0	31.0	28.0	24.0	21.0	28.0	31.0	24.0
**GG**	1.6	3.6	2.0	3.9	3.6	1.4	0.0	6.1	3.6	0.0	1.4	6.1

Allelic and genotypic frequencies of SNPs of the IL7Rα gene in CAP adult according to age and sex. Frequencies are shown as percentages. P values were calculated using the likelihood-ratio Chi-squared test for allelic or genotypic frequencies of each SNP in each group. [a]p value <0.05; [b]p value <0.01; [c]p value <0.001.

Supplementary Table 3

SNP	Genotype	<7 days of illness			≥7 days of illness		
		**n**	**sIL7Rα (ng/ml) Median [IQR]**	**p**	**n**	**sIL7Rα (ng/ml) Median [IQR]**	**p**
**rs6897932**	CC	48	30.41 [23.85-45.64]	<0.0001	22	23.62 [17,61-32.44]	0.28
	CT	35	17.6 [14.45-25.63]		25	20.9 [14.35-35.07]	
	CC	48	30.41 [23.85-45.64]	<0.0001	22	23.62 [17,61-32.44]	<0.0001
	TT	12	10.57 [8.68-15.77]		10	9.21 [8.01-11.58]	
	CT	35	17.6 [14.45-25.63]	0.006	25	20.9 [14.35-35.07]	<0.0001
	TT	12	10.57 [8.68-15.77]		10	9.21 [8.01-11.58]	
**rs987106**	AA	25	27.96 [23.43-45.12]	0.02	13	21.27 [16.98-29.98]	0.83
	AT	47	23.53 [17.07-33.02]		24	21.05 [16.45-35.26]	
	AA	25	27.96 [23.43-45.12]	0.001	13	21.27 [16.98-29.98]	0.03
	TT	23	16.09 [9.53-29.04]		20	12.48 [8.97-26.12]	
	AT	47	23.53 [17.07-33.02]	0.02	24	21.05 [16.45-35.26]	0.02
	TT	23	16.09 [9.53-29.04]		20	12.48 [8.97-26.12]	
**rs3194051**	AA	66	22.37 [14.64-32.00]	0.05	41	17.18 [11.5-22.99]	0.04
	AG	26	26.76 [18.53-45.52]		13	25.25 [15.98-40.02]	
	AA	66	22.37 [14.64-32.00]	0.15	41	17.18 [11.5-22.99]	0.10
	GG	3	29.72 [29.40-40.46]		3	26.41 [25.04-31-35]	
	AG	26	26.76 [18.53-45.52]	0.65	13	25.25 [15.98-40.02]	0.9
	GG	3	29.72 [29.40-40.46]		3	26.41 [25.04-31-35]	

sIL7Rα levels in adults with CAP according to days of illness and the genotypes of the *IL7Rα* gene .
P values were calculated by Mann Whitney test

IQR: interquartile range

Supplementary Table 4

SNP	Genotype	n	IL-7 pg/ml Median [IQR]*
**rs6897932**	CC	79	4.70 [2.72 – 7.14]
	CT	65	5.04 [2.11 – 7.50]
	TT	25	5.74 [2.96 – 9.97]
**rs987106**	AA	40	5.21 [2.72 – 7.67]
	AT	83	4.70 [2.11 – 7.32]
	TT	46	5.04 [2.88 – 7.85]
**rs3194051**	AA	116	5.21 [2.49 – 7.85]
	AG	48	4.19 [2.76 – 6.00]
	GG	5	5.04 [3.54 – 9.97]

Levels of IL-7 in plasma from 144 adults with CAP according to genotypes in SNP rs6897932, rs987106 and rs3194051 of the *IL7Rα* gene . *P>0.05; Mann-Whitney test.
